# Self-reported expressibility predicts communicative success: Open dataset, validation, and simulation

**DOI:** 10.3758/s13428-026-03071-4

**Published:** 2026-06-12

**Authors:** Aleksandra Ćwiek, Susanne Fuchs, Wim Pouw, Šárka Kadavá

**Affiliations:** 1 Center for Language Evolution Studies, Nicolaus Copernicus University in Toruń, Toruń, Poland; 2 Institute of Advanced Studies, Nicolaus Copernicus University in Toruń, Toruń, Poland; 3 Leibniz-Centre General Linguistics, Berlin, Germany; 4 Department of Computational Cognitive Science, Research Center for Cognitive Science and Artificial Intelligence, Tilburg University, Tilburg, The Netherlands; 5 Donders Institute for Brain, Cognition and Behaviour, Radboud University, Erasmusplein 1, 6525HT Nijmegen, The Netherlands; 6 University of Göttingen, Göttingen, Germany

**Keywords:** Expressibility ratings, Experimental semiotics, Referential communication, Modality, Communicative success, Iconicity

## Abstract

Referential communication experiments, in which participants negotiate meaning without conventional language, have provided insights into the origins of language and the modalities that accompanied its early forms. However, these experiments are resource-intensive, limiting sample sizes and the diversity of stimuli. This study provides an empirical validation of expressibility ratings – participants’ subjective judgments about their ability to communicate concepts through different modalities – as an efficient alternative to laboratory-based production experiments. We collected expressibility ratings for 207 concepts across three modalities: gesture-only, vocalization-only, and combined gesture-vocalization. These ratings were empirically validated against a production experiment in which participants attempted to communicate 84 selected concepts. We measured communicative success using binary accuracy (correct/incorrect) and continuous semantic similarity between guesses and targets. Results strongly support the empirical validity of expressibility ratings. Higher expressibility ratings reliably predicted greater guessability (both measures) and required fewer corrective attempts when feedback was provided. Feedback improved exact matches but not semantic similarity. Modality comparisons revealed that gesture-only and combined modalities performed equivalently and both substantially better than vocalization-only. To further showcase the utility of the ratings, we created robust simulations to investigate potential bias in concept selection for stimuli. The analysis examined varying semantic domains and stimulus and participant numbers, finding no systematic bias toward gestural superiority, suggesting robust modality differences. The strong correspondence between expressibility ratings and actual communicative success demonstrates that humans have intuitions of their nonlinguistic communicative abilities. These findings establish expressibility ratings as a methodologically efficient tool for studying modality affordances, enabling larger-scale investigations with enhanced statistical power and broader conceptual coverage.

## Introduction

Human communication represents a remarkable achievement – the ability to create and share meaning through diverse channels. When conventional linguistic systems are unavailable, humans demonstrate creativity in developing ad hoc communication strategies to depict certain meanings. This capacity has interested researchers for decades, with referential communication experiments emerging as a paradigm for investigating how novel meaning systems develop and function.

Referential communication experiments – in which participants negotiate meaning from scratch with a pretended absence of language – form the core of experimental semiotics, a research tradition that investigates how novel communication systems emerge and function (Galantucci & Garrod, 2010, 2011). This approach has enhanced our understanding of language evolution (Erben Johansson et al., 2021; Fay et al., 2014, 2022; Garrod et al., 2010; Hawkins et al., 2023a, 2023b; Kempe et al., 2019; Kirby et al., 2008; Lister et al., 2021; Macuch Silva et al., 2020; Raviv & Arnon, 2018; Selten & Warglien, 2007; Verhoef et al., 2014). They typically involve one participant (the “producer”) attempting to communicate a meaning to another (the “guesser”) using gestures, vocalizations, and their combinations, drawing, or artificial systems. By investigating novel bodily or other expressions of producers and the comprehension by the guesser, we gain insights into the processes through which shared meaning emerges, as well as communicative affordances of different modalities and meanings. By modality affordances, we refer to the differential effectiveness of modalities for successfully communicating different types of meaning, based on the match between modality properties and concept properties.

This paper has the following structure. First, we briefly discuss below which theoretical implications have been drawn from experiments that investigate bodily expressions by the upper limbs and/or the vocal apparatus, i.e., using gestures and/or vocalizations. We further address methodological constraints arising from the labor-intensive nature of these experiments and their potential impact on the results. To overcome these constraints, this paper then offers a lightweight data alternative in the form of ratings validated in a laboratory study. To further test these constraints, we report on a simulation study that investigates the effects of concept choice, number of concepts, and participants. We provide the ratings as an open dataset, allowing further research to benefit from them.

### Modality effectiveness and methodological challenges of their comparison

The theoretical debate about how modality affects communication is primarily concerned with how human gestures and/or vocalizations can express the meaning of concepts. A significant body of research has found that gestures generally outperform vocalizations when participants are asked to communicate without conventional language (Fay et al., 2014, 2022; Zlatev et al., 2017). Even children seem to have no issue with creating a novel gesture language during social interaction (Bohn et al., 2019). Gestures appear particularly effective for representing visual-spatial concepts, allowing for direct iconic mappings between hand movements and physical properties such as shape, size, and motion (Perniss et al., 2015). Finally, ape gestures are said to exhibit greater flexibility than vocalizations (Pollick & Waal, 2007). In the absence of linguistic conventions, this gestural advantage has supported the “gesture-first” hypothesis regarding the origins of language (Arbib et al., 2008; Corballis, 2002; Hewes, 1973).

However, vocalizations also demonstrate considerable iconic potential. Participants can create vocal representations that successfully communicate meanings to naïve listeners, particularly for sound-related concepts or emotional states (Perlman & Lupyan, 2018; Perlman et al., 2015). Cross-linguistic research shows that certain semantic categories are more readily guessed through vocalizations than others, suggesting domain-specific vocal affordances (Ćwiek et al., 2021). The principle – termed modality-congruent expression (Perlman & Cain, 2014; Perniss et al., 2015) or the direct linkage hypothesis (Macuch Silva et al., 2020) – predicts that visual concepts are easier to convey in gesture (direct visual-spatial mapping), while vocal concepts are easier to convey in vocalization (direct acoustic mapping).

At the same time, direct experimental comparisons of the modalities are rather rare (cf. Fay et al., 2014, 2022; Macuch Silva et al., 2020), and varying methodological choices make it difficult to compare findings across different studies. Consider the impact of concept selection: from a vast number of possibilities, researchers make different choices regarding conceptual domains that they focus on, e.g., Fay et al. (2014) used emotions, actions, and objects; Fay et al. (2022) used basic English vocabulary across nouns, verbs, and adjectives; and Macuch Silva et al. (2020) chose visual and auditory stimuli. Experimental designs also diverge in interaction protocols – some permitting communicative exchange between participants (Macuch Silva et al., 2020), others limiting their possibility to interact (e.g., Hawkins et al., 2023a, 2023b; Selten & Warglien, 2007), and some include no interaction at all (e.g., Ćwiek et al., 2021; Fay et al., 2022).

Another important methodological aspect that may have an impact on the research findings and their interpretation are practical constraints: multimodal communication experiments are overall resource-intensive, often requiring long lab sessions and costly data collection. These demands often limit the number of stimuli and participants – choices that not only shape experimental design but also directly impact statistical power and the generalizability of findings.

### Expressibility ratings as an alternative approach

In response to these methodological challenges, we propose self-reported expressibility ratings as an alternative and more resource-efficient approach to study the relationship between modalities, concept affordances, and communicative success. Expressibility^1^ ratings represent participants’ subjective judgments about how effectively they believe they could communicate specific concepts without language while using different modalities (gesture, vocalization, or their combination). Unlike referential-task experiments, which measure actual communicative success between interlocutors, expressibility ratings measure anticipated communicative success based on intuitive judgments.

The methodological advantages of expressibility ratings are substantial. They can be collected efficiently through online platforms, allowing researchers to gather data from larger participant samples across more extensive concept sets than would be feasible in laboratory-based production experiments, but do expressibility ratings actually predict communicative success? This question is not merely methodological, but it also connects to fundamental issues in human cognition and to a broader research tradition examining whether subjective judgments can serve as valid proxies for actual behavior. Research on metacognitive judgments (Koriat, 1997) and social perception (Ambady & Rosenthal, 1992) demonstrates that under certain conditions, people’s assessments can reliably predict behavioral outcomes. However, research on communicative effectiveness specifically shows more mixed results. Speakers systematically overestimate how transparently they communicate (Keysar & Henly, 2002), assuming their intended meanings are more obvious to listeners than they actually are. This “illusion of transparency” appears particularly pronounced in novel or ambiguous contexts where predictable feedback is absent (Inbar et al., 2010; Kahneman & Klein, 2009).

The gap between intuition and actual performance highlights the need to empirically validate expressibility ratings before accepting them as proxies for communicative outcomes. If participants’ judgments about expressibility reliably predict actual guessability, this would provide empirical validation for a methodologically efficient approach for studying modality affordances. It would also demonstrate that humans possess relatively accurate intuitions about their ability to communicate single concepts across different modalities – a finding of considerable theoretical interest to psychology, which has been skeptical about humans’ introspective abilities.

### The aims of this study

This study aims to provide an empirical validation of expressibility ratings as predictors of actual communicative success when expressing single concepts without conventional language. Specifically, we test whether participants’ subjective judgments about how effectively they could communicate concepts through different modalities reliably predict actual guessability in a laboratory-based referential communication task. This addresses a methodological question (can ratings serve as an efficient proxy for resource-intensive production experiments?) and possibly also a theoretical question about metacognitive awareness (do people accurately judge their nonlinguistic communicative abilities?). We collect expressibility ratings for individual lexical items, following standard practice in rating studies across diverse constructs, including iconicity (Winter et al., 2023), age of acquisition (Kuperman et al., 2012), or sensorimotor norms (Lynott et al., 2020). Our validation focuses on single concepts (individual lexical items) rather than more complex communicative structures such as events or narratives. Individual words provide clear, controlled targets for both rating and behavioral validation. By establishing that expressibility ratings predict communicative success, we provide an empirically validated methodology for investigating modality affordances.

To validate expressibility ratings, we must establish that they predict actual patterns of communicative success. This requires examining how guessability differs across modalities in our production experiment. If expressibility ratings reliably predict these patterns, this demonstrates that they capture meaningful aspects of communicative potential across different modalities.

Furthermore, we aim to demonstrate how empirically validated expressibility ratings can also serve as a foundation for simulation-based approaches that investigate how methodological decisions influence research outcomes. As noted, researchers make different choices regarding conceptual domains and specific concepts therein (Ćwiek et al., 2021; Fay et al., 2014, 2022; Lister et al., 2021). For instance, Fay et al. (2014) use concepts from categories, actions, objects, and emotions as stimuli in their experiment investigating modality affordances of gestures versus vocalizations. If actions and objects offer better affordances in the visual modality, it is plausible to wonder how the choice of a category could potentially yield different results. If so, there is a possibility that concept selection can bias results toward particular conclusions about modality effectiveness. Through simulations based on validated expressibility ratings, we can vary concept selections, sample sizes, and semantic domains to quantify how these methodological choices affect the likelihood of observing modality differences (Kadavá et al., 2024). This approach could help unify results that are currently challenging to compare due to different concept selection, varying sample sizes, and methodological differences across studies. For example, Fay et al., (2014, 2022) interpret their findings as supporting gesture as the most effective modality, while Macuch Silva et al. (2020) report that multimodality provides more efficiency compared to gesture, i.e., trial length is substantially shorter in multimodal; but these studies differ substantially in their experimental paradigms and stimuli selection. The proposed framework could help assemble concept sets with balanced modality affordances for future studies and strategically select concepts to test specific hypotheses about particular semantic domains.

However, before expressibility ratings can serve these methodological and theoretical purposes, we must first establish their validity as predictors of actual communicative success. The present study addresses this foundational question by directly comparing expressibility ratings with outcomes from laboratory-based referential communication tasks. Only if ratings reliably predict actual guessability can we confidently use them for simulation studies and methodological evaluations.

To empirically validate expressibility ratings and explore their utility for simulation-based research, we investigate four specific hypotheses regarding expressibility and communicative success, and one additional hypothesis:

- H1: Higher expressibility ratings correspond to higher guessability.

This hypothesis addresses our core validation question: Do participants’ judgments about how expressible a concept would be in a particular modality predict whether that concept is actually guessed correctly when communicated through that modality? A strong positive relationship would suggest that expressibility ratings capture actual ratings in a lab study.

- H2: Concepts with high expressibility ratings require fewer repetitions before being guessed correctly.

In this hypothesis, we examine whether expressibility ratings predict communicative efficiency. When participants attempt to communicate concepts rated as highly expressible, do they require fewer attempts before achieving successful communication compared to concepts rated as less expressible? This relationship would validate expressibility ratings as indicators of communicative efficiency.

- H3: Feedback improves guessability.

This hypothesis addresses how communicative adaptation influences outcomes in referential tasks. Does providing feedback, i.e., letting participants know what their partner’s guess was, enhance subsequent guessability? This question has both methodological implications for experimental design and theoretical relevance for understanding how communicative signals evolve through interaction.

- H4: There exists a hierarchy in modality guessability, with combined signals surpassing gesture-only, and gesture-only surpassing vocalization-only.

This hypothesis examines whether our study replicates previously observed patterns regarding modality effectiveness (such as Fay et al., 2022; Macuch Silva et al., 2020). Establishing how modalities differ in actual guessability is necessary for validating expressibility ratings: if ratings predict these empirical patterns, this demonstrates they capture meaningful differences in communicative potential across modalities.

Additionally, we investigate how concept selection influences conclusions about modality dominance (concretely, gesture versus vocal), using expressibility ratings to simulate experiments with varying concept sets and sample sizes. This simulation-based approach demonstrates the utility of the expressibility ratings for further methodological evaluations.

- H5: The likelihood of observing a reliable difference between gesture and vocal expressibility is influenced by experimental design parameters, namely the number of participants (i.e., observations), the number of concepts (i.e., stimuli), and the semantic domain of stimuli.

This simulation approach allows us to assess whether findings about modality effectiveness are likely to replicate across different experimental designs or potentially the choice of specific concept selections. The full rationale behind the simulations is elaborated in Section “Simulation design rationale”.

To test H1-H4, we compare expressibility ratings collected in an online study with actual outcomes from a laboratory experiment using a subset of the larger set of concepts that were rated online. We assess guessability both as a binary variable (correct/incorrect) and as a continuous measure of semantic similarity between the guess and the target concept. This dual approach acknowledges that communication operates on a spectrum rather than being simply successful or unsuccessful. To test H5, we design a set of simulated experiments with varying sample size and concept choice and analyze the simulated data in terms of the likelihood of observing a difference between gesture and vocal modalities.

Hypotheses 1–4, the research aims, and the planned analyses were pre-registered on November 24, 2024, on AsPredicted (#200,596): https://aspredicted.org/kmry-vx5s.pdf.

## Method

### Expressibility ratings

We collected expressibility ratings to capture participants’ intuitions about how effectively a set of 207 concepts could be communicated using three distinct modality conditions: gesture, vocalization, and a combination of gesture and vocalization. The 207 concepts were selected from various sources, including the Leipzig-Jakarta List (Tadmor et al., 2010) and sensorimotor norms (Lynott et al., 2020), ensuring a diverse range of meanings across multiple semantic dimensions. All concepts were single lexical items from a range of parts of speech rather than complex events or multi-word expressions, ensuring that expressibility judgments pertained to basic, atomic meanings.

Participants, all native Dutch speakers recruited from a university participant pool, provided expressibility ratings on a continuous scale from – 1 to 1 (later converted to 0 to 1), where −1 indicated “very bad” expressibility and 1 indicated “very good.” Each participant rated 60 concepts, with 20 assigned to each of the three modalities (gesture, vocalization, and multimodal). The assignment of concepts and modalities was randomized so that every concept received ratings in all three modalities from multiple raters. Participants were instructed to imagine how well they could communicate each concept using only gestures with their hands and/or body (no vocalization), only non-linguistic vocalizations (no gestures), or a combination of both without the use of language.

The experiment was designed in PsychoPy2 (Peirce et al., 2019) and hosted online by Pavlovia. After we provided detailed instructions for the modality being rated, participants were presented with individual concepts. Ratings were recorded using a slider interface, and the order of concepts and modalities was fully randomized to control for order effects. Before beginning the main rating session, participants completed practice trials to familiarize themselves with the task and rating scale.

To ensure data quality, we excluded ratings with response times more than three standard deviations from the mean (1% of the data). After exclusions, the participant pool consisted of 215 individuals (1 diverse, 41 males, 173 females; average age 20, range 18–68), yielding 12,678 data points. On average, each concept received between 16 and 23 ratings.

For analysis, we employed four Bayesian hierarchical zero–one inflated beta models. Each model, with four chains, was run for 4000 post-warmup samples. All models converged well (Rhat = 1.00). We used leave-one-out cross-validation to select the best-fit model. The chosen model treated expressibility as a function of modality (with three levels, where the combined modality served as the baseline) and included by-concept intercepts and slopes for modality in all model components (φ, zoi, coi). From the final model, we extracted posterior samples for each concept’s modality-specific expressibility values, ultimately obtaining posterior means used in this study. The procedure was identical to Kadavá et al. (2024), but applied to a different population.

### Production experiment

The production experiment (pre-registered in Kadavá et al., 2023) tested how effectively participants could communicate 84 concepts using gesture, vocalization, or a combination of both. The concepts were systematically selected from the larger 206-concept pool rated for expressibility (one concept from 207 had to be excluded from this data due to an error in translation). This selection balanced experimental feasibility (manageable session length and participant number given the 3 modalities 2 × feedback conditions design) with methodological quality. We excluded bodily concepts as they would tend to be performed indexically (e.g., leg, arm), concepts with very low expressibility (below mean −1 SD per modality), as they would impose too large a difficulty for the performers, and then selected top-expressible concepts ensuring balanced, non-overlapping sets per modality (28 concepts each). Full selection procedure and rationale are documented in the pre-registration of the production experiment (Kadavá et al., 2023).

Participants of the production experiment were Dutch native speakers and were recruited in dyads through Radboud University’s participant recruitment platform. These participants were different from those of the expressibility rating study. The final sample consisted of 142 participants from 71 dyads (1 diverse, 1 non-binary, 1 not specified, 24 males, 113 females; average age 20, range 17–27). One dyad was excluded due to consent withdrawal. Each dyad member was randomly assigned to serve as a performer or a guesser in the beginning. The performer produced the target concept using only the assigned modality, without using language. The guesser attempted to guess the intended concept. Roles alternated within each session, ensuring that each participant both performed and guessed an equal number of concepts across all three modalities.

The experiment was conducted in two parts. In part 1, there was no feedback: after each production, the guesser typed their answer, but neither performer nor guesser learned whether it was correct. In part 2, feedback was introduced: the guesser was informed if their guess was correct, and the performer saw the guesser’s response. If the guess was incorrect, the performer could try to improve their performance up to two more times, allowing us to examine whether anticipated feedback enhanced guessability. The order was fixed (no-feedback first, feedback second) as pre-registered (Kadavá et al., 2023), driven by our research question about how feedback transforms initial communicative attempts. Part 1 establishes baseline performance; Part 2 examines adaptation through feedback. Performance in part 2 could reflect both feedback effects and general task familiarity.

Each participant performed and guessed 21 concepts (7 per modality) in each part, for a total of 84 concepts per dyad. Concept-to-modality assignments were randomized, and each concept appeared only once per dyad to prevent learning effects. In the gesture condition, performers used only hand and body movements; in the vocalization condition, they used only non-linguistic vocal sounds; and in the combined condition, they could use both. The use of language was not allowed.

The experiment took place in a controlled laboratory setting. Performers were standing to enable movement of the whole body rather than limiting productions to manual gestures. This design choice aligns with experimental semiotics research on pantomime and novel communication systems, demonstrating that standing position facilitates whole-body movement, whereas seated position promotes more restricted manual gesturing (Placiński et al., 2023; Zlatev et al., 2024). Additionally, the standing position enabled measurement of postural dynamics, which was part of the broader research program (see Kadavá et al., 2023, 2025). Performers and guessers were separated by a one-way screen such that only the guesser could see the performer. Additionally, a headset microphone and headphones were used in vocal and multimodal blocks to ensure good audio discriminability, while in the gesture block, the guesser switched to noise-canceling headphones to prevent any vocal interference. Performers indicated the start and end of each production with a predefined gesture. Guesser responses were recorded via keyboard.

### Analysis: Validation of expressibility

The data, scripts, and models are available in the GitHub repository: https://github.com/sarkadava/FLESH_Expressibility_vs_guessability.

Data from 71 dyads were included in the analysis. There were 8989 data points: 3401 in vocalization, 2799 in gesture, and 2789 in combined (the discrepancy in the number is due to the design allowing for repetitions). There were 2968 data points in experiment part 1 (994 vocal, 980 gesture, 994 combined) and 6021 in experiment part 2 (2407 vocal, 1819 gesture, 1795 combined).

All analyses were conducted using Bayesian hierarchical models in the brms package (v. 2.22.0; Bürkner, 2017) in R (v. 4.4.1; R Core Team, 2024). Data preprocessing, manipulation, and visualization were handled using the tidyverse (v. 2.0.0; Wickham et al., 2019) suite of packages. Model comparison was conducted via the loo package (v. 2.8.0; Vehtari et al., 2024). Results were processed and summarized using emmeans for effect extraction (v. 1.11.0; Lenth et al., 2024), HDInterval for credible interval calculations (v. 0.2.4; Juat et al., 2022), and tidybayes for posterior extraction and manipulation (v. 3.0.7; Kay & Mastny, 2024).

#### Variables

The primary dependent variable (guessability) was operationalized in two ways: as a binary variable (correct/incorrect) and as a continuous measure of semantic similarity (cosine similarity) between the guesser’s answer and the target concept. Cosine similarity was computed using Dutch word embeddings from ConceptNet (Speer et al., 2017), with values originally ranging from − 1 to 1 and then transformed to 0–1 to accommodate the requirements of the model family. It is important to note that concepts originally ranging from − 1 to 0 relate to the degree of dissimilarity (i.e., concepts deemed not confusable with each other), and those ranging from 0 to 1 relate to the degree of similarity; in the transformed scale, they are represented as 0 to 0.5 and 0.5 to 1, respectively. Some pairs (*N* = 130, ~ 2% of the data) were not available in ConceptNet, and for those, we asked 16 independent raters to assess the perceived similarity. Validation data from 14 independent raters revealed a strong correlation (*r* = 0.73, BF10 = 3.728 × 10^18^) between perceived similarity and cosine similarity. For seven of the answers, no cosine similarity could be measured because they were not informative (e.g., “I don’t know”, “no idea”).

#### Modeling

We first fit an intercept-only model to assess baseline guessability. To test our hypotheses, we included the posterior mean expressibility ratings (from the earlier rating study) as predictors of guessability outcomes (H1) and the number of repetitions (H2, tested only on part 2). We also tested guessability as a function of the experiment part to test whether feedback improves guessability (H3). Finally, we tested whether guessability differed by modality (H4), with a hypothesized hierarchy: combined > gesture > vocalization. All models included random intercepts by concept and by dyad to account for group-level variation. This differs from our pre-registration, which included a random intercept for dyad with nested participants. However, we found that such an approach dramatically increased the computational effort, even when adjusting all control settings. This was especially problematic when modeling cosine similarity with the zero–one-inflated beta (ZOIB) family, which was the preferred family due to data distribution. Thus, we decided to compute the intercept-only models of cosine similarity using various settings: including the nesting and priors for family-specific parameters (ZOIB priors), including the nesting but not the ZOIB priors, and finally including neither the nesting nor the ZOIB priors. Our model comparisons using leave‐one‐out (LOO) cross-validation − a method that estimates out-of-sample predictive performance by iteratively leaving out each observation and predicting it from the model fitted to the remaining data (Vehtari et al., 2017) − revealed that the model with explicit ZOIB priors and fully nested random effects performed much worse than our simpler models. When we compared the more complex nested model (estimated predictive accuracy ELPD ≈ − 896.62 ± 93.6) with a simpler model that removed the extra dyad/participant-nesting and only included a group-level effect by dyad (ELPD ≈ − 902.57 ± 93.7), the difference in predictive performance was very small at only about 5.94 (SE 0.13) points. While we do not apply an arbitrary cutoff point in comparing the ELPD values, in accordance with Sivula et al. (2025), we can interpret these values as suggesting that the added complexity did not improve the model in a meaningful way. In light of these results, we have decided to proceed without the explicit ZOIB priors and without the additional nesting. The simpler model not only avoids potential overparameterization and convergence issues but also yields nearly identical predictive performance.

We modeled binary guessability using a Bernoulli family with logit link and cosine similarity using a zero–one inflated beta family, which was necessary due to the bimodal distribution. For the number of repetitions, we used a Poisson model. We used weakly informative priors following best practices in Bayesian modeling (McElreath, 2018). These priors gently regularize parameters toward zero while allowing substantial deviations supported by data, improving computational efficiency without strongly constraining inference. The following priors were used: normal (0, 2.5) for intercepts, normal (0, 1) for regression coefficients, and normal (0, 2) for random effect standard deviations across all models. For the Bernoulli model, the intercept prior operates on the logit scale, while for the Poisson model, it operates on the log scale. The ZOIB model used these same priors for the mean component (μ), with default priors for the precision (φ), zero-or-one inflation (zoi), and one inflation (coi) parameters. All models were run with 10,000 iterations (4000 warm-up iterations) and four chains, resulting in a total of 24,000 post-warm-up samples. Intercept-only models achieved Rhat ≤ 1.01, and all main models achieved Rhat = 1.00, the latter indicating excellent convergence.

### Analysis: Simulations

**Fig. 1:**
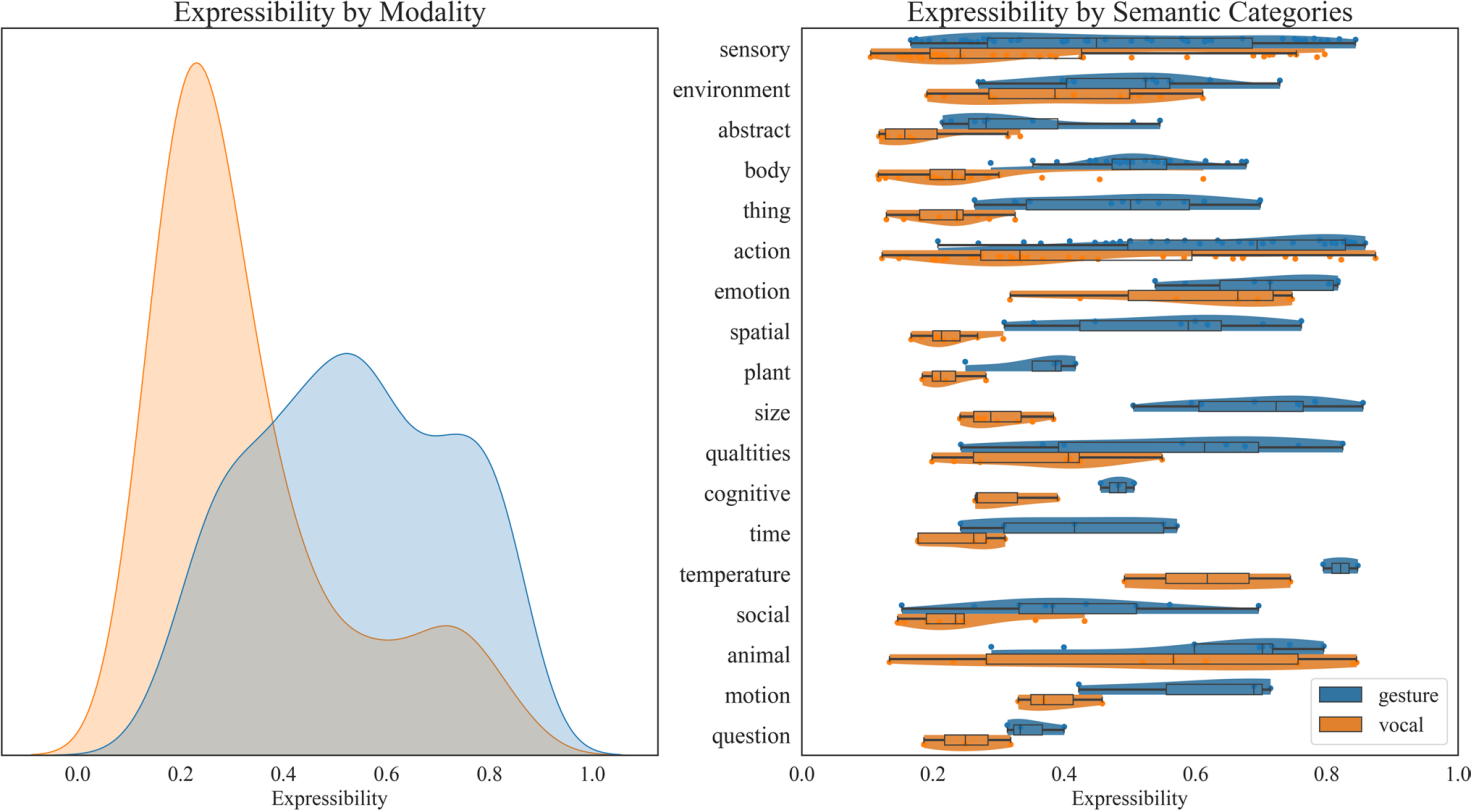
Distribution of expressibility for vocal (*orange*) and gestural (*blue*) modalities. The *left plot* shows distributions by modality, the *right plot* shows distributions by modality and semantic category

To showcase the utility of expressibility ratings, we use the posterior distributions of expressibility for each concept modeled in Section *Expressibility ratings* to simulate experiments with varying experimental design parameters. Figure 1 shows the posterior distributions of expressibility values within concept categories and overall.

As can be seen, the datapoints for the 207 concepts show that gesture is generally rated higher in expressibility than vocalization (mean expressibility for gesture = 0.535, mean expressibility for vocalization = 0.337). However, the substantial overlap between vocal and gestural expressibility within some categories suggests that observed differences between modalities may be driven partly by concept selection rather than solely by inherent modality constraints, highlighting the importance of stimulus choice in experiments testing modality affordances. In other words, there may be a bias to select concepts that specifically drive a dominance of gestures over vocalizations. To test this possibility systematically, we designed five simulations that manipulate concept selection strategies.

#### Simulation design rationale

Each simulation tests a different hypothesis about how concept selection affects the observed modality differences.

##### Simulation 1: Baseline (random selection across all categories)

In this simulation, concepts were randomly selected across all 18 available semantic categories (action, abstract, time, animal, emotion, body, thing, sensory, environment, plant, size, spatial, social, qualities, temperature, motion, cognitive, and question), regardless of which category was represented how many times. This simulation establishes a baseline for modality differences when concepts are sampled without semantic constraints. If modality differences are robust across semantic domains, we expect a low proportion of inconclusive results. Conversely, if modality differences depend on which categories happen to be sampled, we expect high variability in Bayes factors and effect sizes across iterations.

##### Simulation 2: Replication of prior work (three established semantic categories)

In this simulation, concept selection was constrained to three specific categories: action, object, and emotion. This simulation tests whether the established findings of gesture superiority from Fay et al. (2014) is replicable. If these specific categories systematically favor gesture over vocalization, we expect higher proportions of conclusive results and larger, more consistent effect sizes compared to other simulations. Conversely, if these categories are representative of typical modality differences, outcomes should be similar to the baseline (Simulation 1).

##### Simulation 3: Category-driven variability (combination of three random categories)

In this simulation, concepts are selected from three randomly determined semantic categories from all 18 available categories. This simulation assesses how much variability in modality differences arises purely from which semantic categories happen to be included. If different combinations of categories yield substantially different proportions of conclusive results and effect sizes, this demonstrates that experimental outcomes depend on which categories researchers choose to sample. We expect greater variability in outcomes across iterations compared to Simulation 2.

##### Simulation 4: Replication of prior work (morphological categories)

In this simulation, concepts’ selection is constrained to three morphological categories − nouns, adjectives, and verbs − following Fay et al. (2022). The simulation tests whether organizing concepts by grammatical class rather than semantic category reduces selection bias. If morphological categorization balances modality affordances differently than semantic categorization (Simulation 2), we may observe different proportions of conclusive results and effect sizes. Specifically, if morphological categories inadvertently sample across both gesture-favoring and more vocally-expressive semantic domains, this could result in lower proportions of conclusive results compared to Simulation 2, particularly in underpowered conditions.

##### Simulation 5: Vocal-favoring category (sanity check)

In this simulation, we test the most extreme case by sampling exclusively from a category assumed to favor vocalization. We chose category emotion for this reason, because, first, emotional states have been shown to be favoring vocal modality (Perlman & Lupyan, 2018), and second, as seen in Fig. 1, the median of vocal expressibility is the closest to gesture from all categories. If gesture superiority in typical experiments is partly driven by sampling from strongly gesture-favoring categories, we expect a substantially higher proportion of inconclusive results and smaller effect sizes in this simulation compared to the others, particularly Simulation 2.

By comparing these five simulation strategies, we can determine: whether the standard finding of gesture superiority holds across different sampling approaches and which selection strategy produces the most stable and generalizable results.

#### Simulation parameters

In addition to varying concept selection strategy, we manipulated the number of concepts (12, 18, 24) and the number of participants per concept (10, 15, 20) in Simulations 1–4, as these factors likely affect the statistical power of the analysis. Given that Simulation 5 serves more as a sanity check, and it already has a low number of concepts available, we ran only one simulation with 10 concepts and 20 participants. This yielded 3 × 3 × 4 + 1 = 37 distinct simulation scenarios (3 concept numbers × 3 participant numbers × 4 selection strategies + 1 sanity-check simulation). For each scenario, we ran 100,000 iterations, where each iteration simulated one complete experiment comparing gesture and vocal expressibility.

Within each iteration, concepts were randomly selected according to the simulation’s selection strategy. For each selected concept, expressibility ratings for both modalities were drawn from the posterior predictive distribution matrix. This approach preserves the uncertainty in our expressibility estimates and reflects realistic experimental variability.

The simulations were conducted using a custom Python script provided in the GitHub repository: https://github.com/sarkadava/FLESH_Expressibility_vs_guessability.

#### Statistical analysis

In the subsequent statistical analysis, we analyzed each simulated experiment using a Bayesian alternative to the independent *t* test, comparing posterior distributions of vocal versus gestural expressibility ratings to determine whether there was a reliable difference between modalities. For each simulation type, we computed several descriptive parameters across all 100,000 iterations. We calculated the Jeffrey–Zellner–Siow (JZS) Bayes factors to assess evidence for differences between gesture and vocal modalities, using the default Cauchy scale factor for computing the Bayes factor set to default 0.707 (medium). For each simulation type, we extracted the mean and standard deviation of Bayes factors and the percentage of experiments with evidence above anecdotal levels (BF > 3). For each simulation type, we also randomly sampled one experiment and drew 2000 posterior samples to extract the mean and standard deviation of the modality difference (gesture minus vocal) and the mean and standard deviation of the corresponding standardized effect size (Cohen’s *d*). These metrics allow us to assess not just whether modality differences exist on average, but how reliably different selection strategies detect these differences and how much the magnitude of effects varies depending on concept selection.

## Results

### Validation of expressibility

We begin by describing two intercept‐only (null) models, then present results for each hypothesis, for H1, H3, and H4, clarifying how the binary vs. cosine similarity metrics differ: the former captures whether a guess matches exactly, while the latter indicates how semantically close the guess is even when incorrect. The combined results for intercept-only, H1, H3, and H4 models are given in Table 1.

**Table 1:** Combined results for intercept-only, expressibility (H1), feedback (H3), and modality (H4) effects on guessability measures (Bernoulli for binary guess, and zero–one-inflated beta for cosine similarity)

	Parameter	Estimate [95% CrI]	Comments
INTERCEPT-ONLY MODELS			
Null (binary)	Intercept (log-odds)	− 0.74 [− 1.02, − 0.46]	~ 32% correct overall
	Random effects (SD)	concept = 1.29; dyad = 0.17	
Null (similarity)	Intercept (beta logit)	0.65 [0.58, 0.72]	~ 77% similarity overall
	zoi intercept	− 0.73	
	Random effects (SD)	concept = 0.30; dyad = 0.03	
HYPOTHESIS 1: EXPRESSIBILITY → GUESSABILITY			
H1 (binary)	Intercept	− 5.34 [− 5.73, − 4.95]	~ 0.5% correct at expr = 0; ~ 27% correct at expr = 0.60; ~ 88% correct at expr = 1.00
	Expressibility slope	+ 7.30 [6.80, 7.81]	
H1 (similarity)	Intercept (beta logit)	0.07 [− 0.00, 0.15]	~ 0.60 sim at expr = 0; ~ 0.75 sim at expr = 0.60; ~ 0.88 sim at expr = 1.00
	zoi intercept	− 0.73	
	Expressibility slope	+ 1.02 [0.93, 1.11]	
HYPOTHESIS 3: FEEDBACK → GUESSABILITY			
H3 (binary)	Intercept (part 1)	− 0.77 [− 1.07, − 0.48]	~ 30% correct in part 1; ~ 33–34% correct in part 2
	Part 2 effect	+ 0.18 [0.07, 0.29]	
H3 (similarity)	Intercept (part 1)	0.65 [approx. from null]	~ 0.655 sim in part 1; ~ 0.66 sim in part 2
	zoi intercept	− 0.73	
	Part 2 effect	+ 0.01 [− 0.02, 0.03]	
HYPOTHESIS 4: MODALITY → (COMBINED > GESTURE > VOCAL)			
H4 (binary)	Intercept (combined)	− 0.14 [− 0.47, 0.19]	~ 46% correct combined; ~ 45% correct gesture; ~ 12% correct vocal
	gesture vs. combined	− 0.03 [− 0.16, 0.09]	
	vocal vs. combined	− 1.85 [− 1.99, − 1.71]	
H4 (similarity)	Intercept (combined)	0.80 [0.74, 0.87]	~ 0.69 sim combined; ~ 0.69 sim gesture; ~ 0.63 sim vocal
	zoi Intercept	− 0.73	
	gesture slope	− 0.01 [− 0.05, 0.02]	
	vocal slope	− 0.29 [− 0.32, − 0.26]	

#### Overall results from production experiment (intercept-only models)

We first ran two baseline models, each with random intercepts for concept and dyad. In the Bernoulli (binary) model, the average log-odds success was − 0.74 (95% CrI: [− 1.02, − 0.46]), which translates to a 32% overall chance of a correct guess pooling across all modalities (95% CrI: [26%, 39%]). There was substantial variation between concepts (SD = 1.29), suggesting that some concepts were much easier to guess than others. The differences between dyads were smaller (SD = 0.17). In the second model, which used a zero–one inflated beta distribution to estimate similarity between the guess and the target meaning, the logit-scale intercept for the main (beta) part of the model was 0.65 (95% CrI: [0.58, 0.72]), and the intercept for the zero–one inflation part was − 0.73. These values correspond to an average cosine similarity of about 0.77. Again, we found noticeable variability across concepts (SD = 0.30), while variation across dyads was low (SD = 0.03).

From this, we see that using the two measures can potentially yield very different results. While the overall posterior probability of a perfect guess is at 32%, the overall posterior probability of a guess based on the cosine similarity is at 0.77. However, it is important to note that our cosine similarity values were normalized to a 0–1 scale from their original range, which included negative values. In this normalized scale, values below 0.5 correspond to what were originally negative cosine similarities (indicating vectors pointing in more *dissimilar* directions), while values above 0.5 correspond to positive similarities, with the degree of similarity increasing toward 1. As such, higher similarity scores are expected, but they do not necessarily imply better guessing performance – they are a feature of the scale itself.

#### Hypothesis 1

According to Hypothesis 1, items rated higher in expressibility should be guessed more accurately. When we added expressibility to the binary model, the intercept was − 5.34 (95% CrI: [− 5.73, − 4.95]), meaning that at zero expressibility, the chance of a correct guess was extremely low, only about 0.5%. The slope for expressibility was + 7.30 (95% CrI: [6.80, 7.81]), indicating a strong positive effect. For example, with an expressibility score at 0.50, the predicted probability of a correct guess rose to ~ 23%, and with an expressibility score of 0.60, the probability increased to ~ 27%. The posterior probability that this slope exceeds zero was effectively 100%, providing strong support for Hypothesis 1 based on binary guesses.

**Fig. 2:**
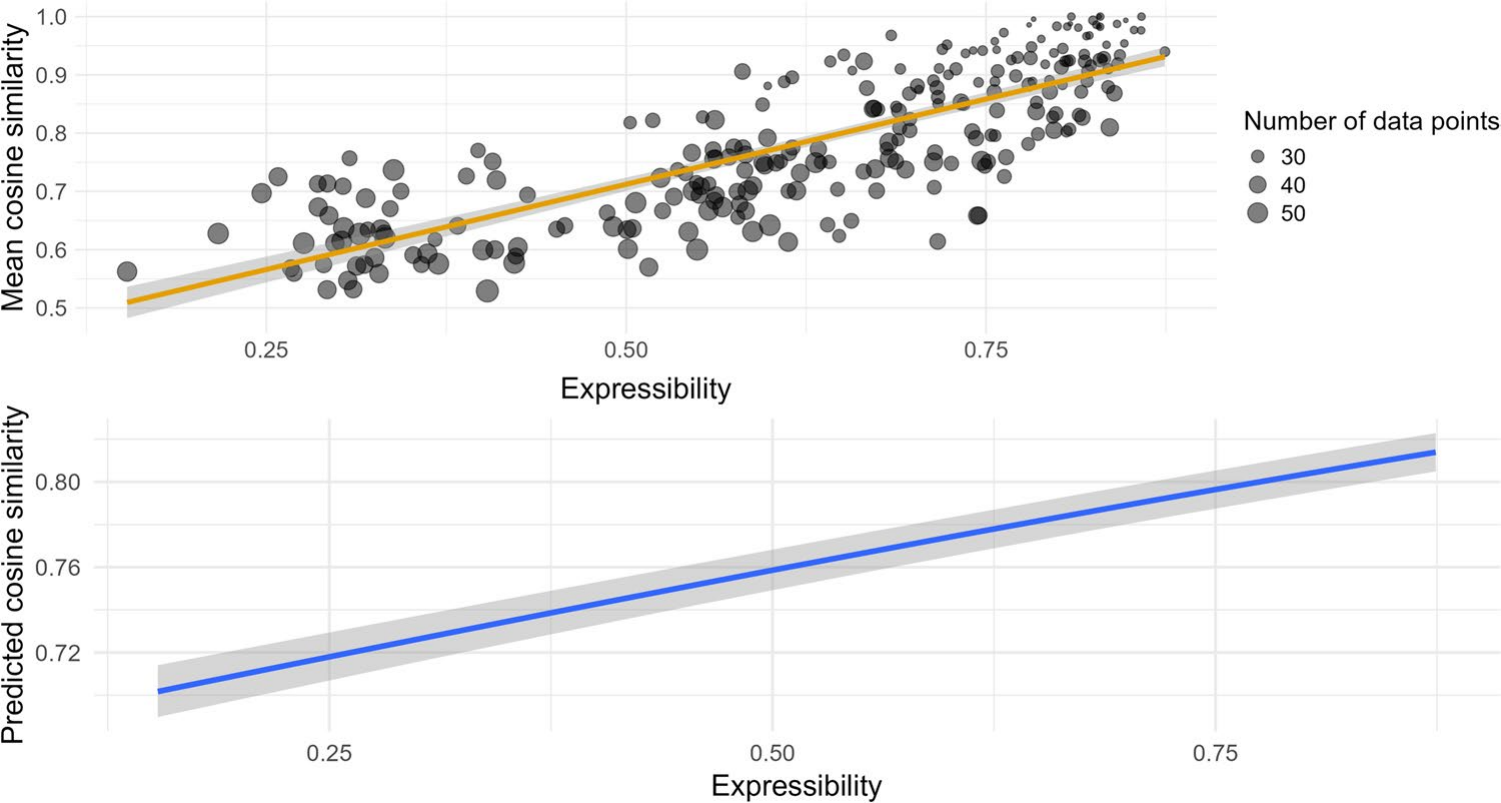
*Top* The average cosine similarity for each unique expressibility value. The size of the *dots* represents the number of original observations contributing to each averaged point. *Bottom* Model-predicted cosine similarity as a function of expressibility. The plot shows the median cosine similarity along with uncertainty bands, illustrating that higher expressibility is associated with a substantially increased cosine similarity

We also looked at the gradient measure of guessability, the cosine similarity. In this model, the intercept was 0.07 (95% CrI: [− 0.00, 0.15]) on the logit scale, and the slope for expressibility was + 1.02 (95% CrI: [0.93, 1.11]). Combined with the same zero–one inflation intercept (− 0.73), this means that more expressible concepts showed higher similarity scores. For example, at an expressibility score of 0.60, the predicted cosine similarity approached 0.75. This shows that higher expressibility boosts both the probability of a binary guess and the cosine similarity. Figure 2 illustrates this effect by showing average raw cosine similarity for each unique expressibility value alongside model-predicted cosine similarity as a function of expressibility.

#### Hypothesis 2

Hypothesis 2 predicts that, when feedback is introduced (in part 2 of the experiment), more expressible items should require fewer corrective attempts to reach understanding. The results are summarized in Table 2.

**Table 2:** Poisson model for hypothesis 2 (higher expressibility leads to fewer repetitions, only in experiment part 2)

Parameter	Estimate [95% CrI]	Overall comments
Intercept (log scale)	+ 0.48 [0.35, 0.61]	~ 1.62 repetitions at expr = 0; each + 1 expr means × 0.20 fewer repetitions; ~ 0.33 repetitions at expr = 1.0
Expressibility slope	− 1.59 [− 1.79, − 1.38]	
Random Effects (SD)	concept = 0.23; dyad = 0.02	

**Fig. 3:**
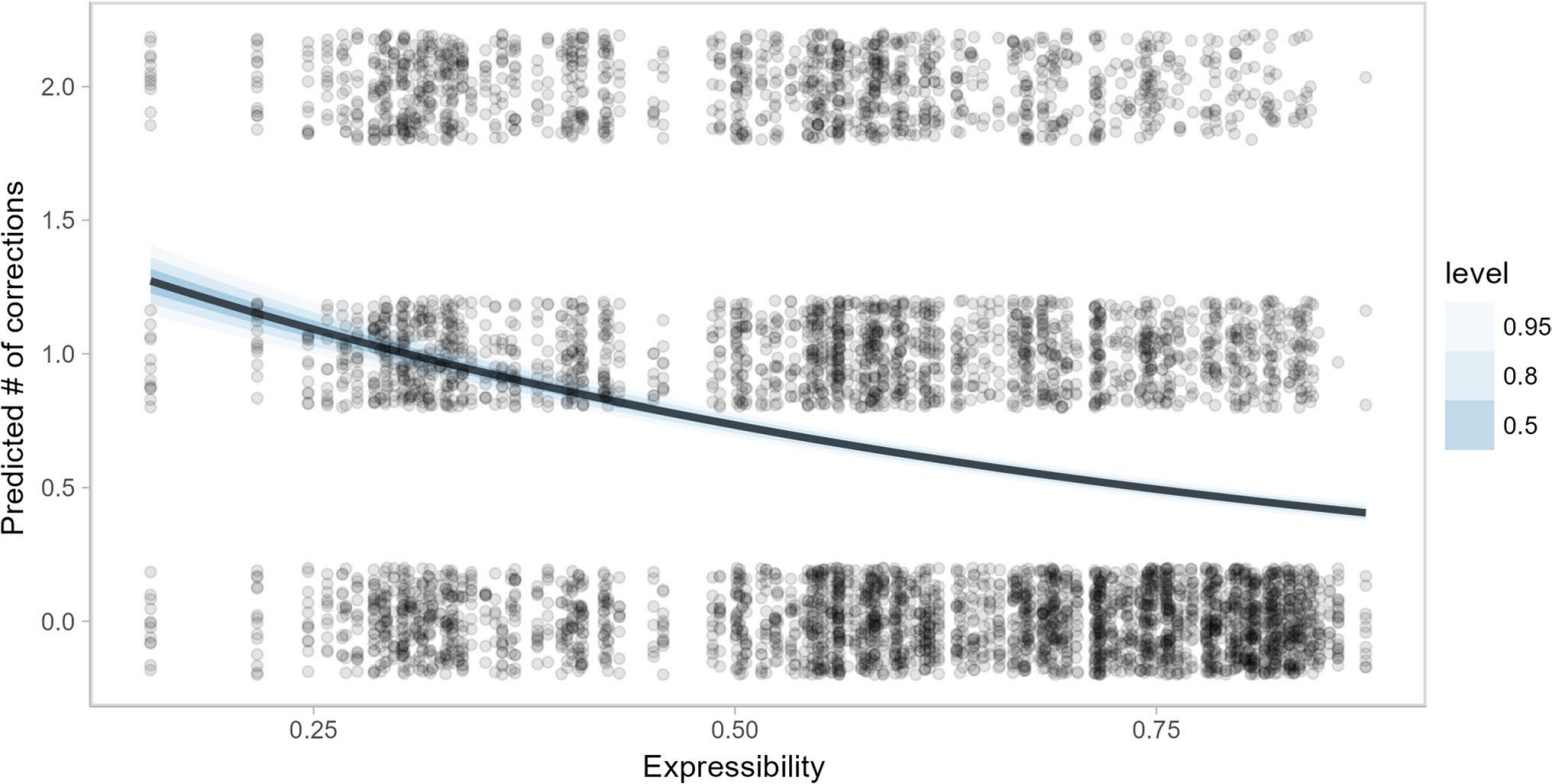
Model-predicted probability of a correction (0 − 2) as a function of expressibility. The plot shows the median predicted probability along with uncertainty bands, illustrating that lower expressibility is associated with more corrections

We fit a Poisson model for the number of correction trials before success. The intercept was + 0.48 (95% CrI [0.35, 0.61]) on the log scale, meaning that for very low-expressibility concepts, about 1.62 correction attempts were needed on average. The slope for expressibility was − 1.59 (95% CrI: [− 1.79, − 1.38]), meaning that each unit increase in expressibility reduced the number of corrections by about 80% (since exp(− 1.59) ≈ 0.20). In concrete terms, the predicted corrections dropped from ~ 1.62 at expressibility = 0.00 to ~ 0.33 at expressibility = 1.00 (see Fig. 3). The posterior checks strongly support this negative relationship, confirming that items with higher expressibility ratings require fewer repairs.

#### Hypothesis 3

Hypothesis 3 states that guessers should become more accurate once feedback is introduced. A Bernoulli model with “experiment part” as a predictor showed an intercept of − 0.77 (95% CrI: [− 1.07, − 0.48]), corresponding to about 30% accuracy in experiment part 1. Moving to experiment part 2 added + 0.18 (95% CrI: [0.07, 0.29]) on the log‐odds scale, increasing accuracy to about 33–34%. Although the improvement is modest, the posterior probability that feedback improves perfect guesses is near 100%, providing credible support for Hypothesis 3.

**Fig. 4:**
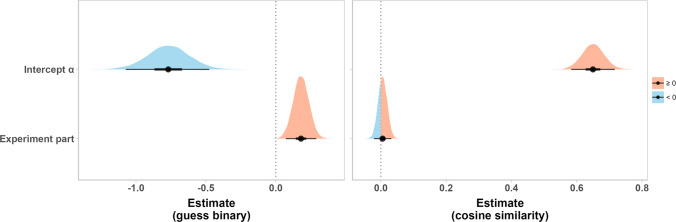
Posterior estimates of the fixed effects showing the median (*dot*), 50% (*thick line*), and 95% (*thin lines*) quantiles. Also showing 0 as the *vertical dotted line* and negative (*blue*) vs. positive (*red*) values. The *left panel* shows the reliable effect of experiment part in the binary guess model as the posterior distribution of experiment part lies reliably on one side of the dotted line (i.e., 100% probability of feedback improving the binary guess); the *right panel* shows no effect of experiment part in the cosine similarity model because the posterior distribution of experiment part includes the 0 crossing point, meaning that the effect of feedback is not reliably positive or negative

However, the zero–one inflated beta model for cosine similarity found no meaningful change: the slope was close to + 0.01 (95% CrI: [− 0.02, 0.03]), with predicted similarity scores staying around 0.65–0.66. This suggests that feedback helps participants more often find exact labels, but does not improve the semantic closeness of near-miss guesses; the difference is illustrated in Fig. 4.

#### Hypothesis 4

**Fig. 5:**
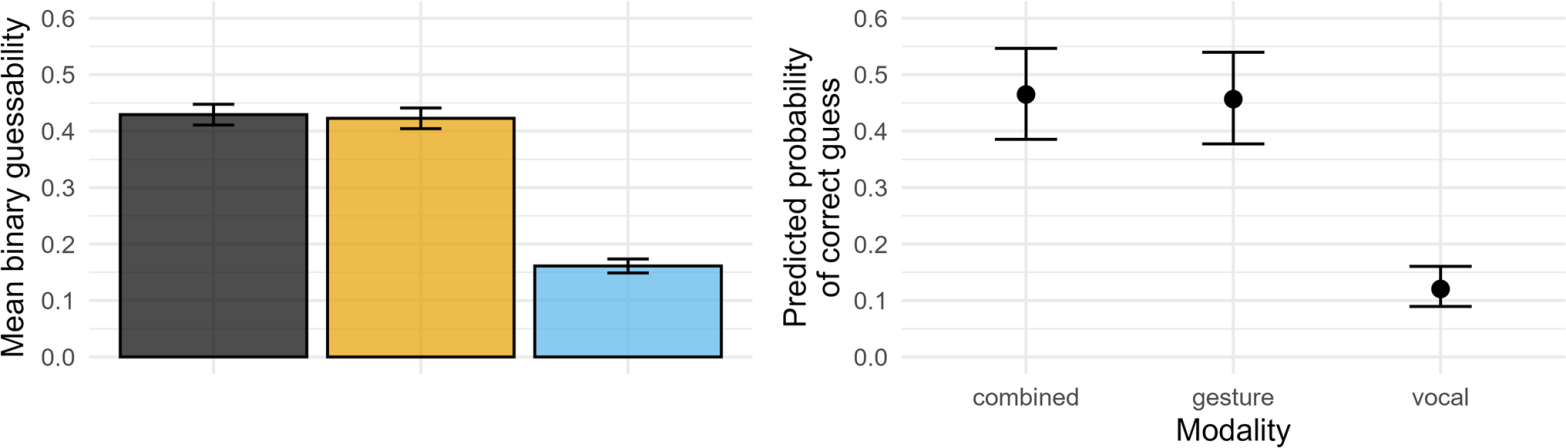
*Left* Raw mean binary guessability by modality. *Right* Model-predicted probability of a correct guess by modality. The plot shows the median predicted probability along with uncertainty bands

Hypothesis 4 predicts that the combined‐signal (gesture and vocalization) condition outperforms gesture alone, and gesture alone outperforms vocalization alone. Using the combined modality condition as baseline, the intercept of the binary guess model was − 0.14 (95% CrI: [− 0.47, 0.19]), corresponding to about 46% accuracy. Gesture was nearly identical to combined (slope =  − 0.03), whereas vocalization performed much worse than combined (slope =  − 1.85, 95% CrI: [− 1.99, − 1.71]), reducing accuracy to about 12%. Pairwise comparisons showed no reliable difference between combined and gesture conditions with a mean difference = 0.01, 95% CrI: [− 0.02, 0.03], and a posterior probability that combined is better than gesture* p*(Combined > Gesture) = 70%. In contrast, both combined and gesture outperformed vocalization with mean differences of 0.34 (95% CrI: [0.30, 0.39]) and 0.34 (95% CrI: [0.29, 0.38]), respectively, and posterior probabilities of 100% for both comparisons. Figure 5 shows the raw values alongside posterior predicted probabilities of a perfect guess.

In the cosine similarity model, the pattern was similar. The combined modality achieved about 0.69 cosine similarity, gesture was statistically indistinguishable (slope ≈ − 0.01), and vocalization reduced similarity to about 0.63 (slope =  − 0.29). Again, pairwise comparisons showed no reliable difference between combined and gesture conditions (mean diff. = 0.00, 95% CrI: [0.00, 0.01],* p*(C > G) = 76%), while vocalization was credibly worse than the other two conditions (combined vs. vocal: mean diff = 0.06, 95% CrI: [0.06, 0.07],* p*(C > V) = 100%, and gesture vs. vocal: mean diff. = 0.06, 95% CrI: [0.06, 0.07],* p*(G > V) = 100%).

### Simulation study: Hypothesis 5

The overall results of all Bayes tests for all performed simulations show that in the majority of the experiments within a simulation, we find decisive evidence for the claim that gesture and vocal expressibility are different (BF > 3). This claim is based on the high percentages of results yielding Bayes factors above 3, indicating decisive evidence favoring the alternative hypothesis over the null hypothesis of no modality difference. The percentage of conclusive results is above 90% in the majority of cases. We do see small, yet varying patterns across the simulations. For instance, Simulation 2 (action-object-emotion selection) yields above 98% of conclusive results for every combination of number of concepts and participants, while in all other simulations the percentages are lower, specifically for those that are ‘underpowered’ (i.e., small number of participants and concepts). The most notable drop of percentage of conclusive results is for Simulation 5 (vocal-favoring modality), and that to 85%.

**Fig. 6:**
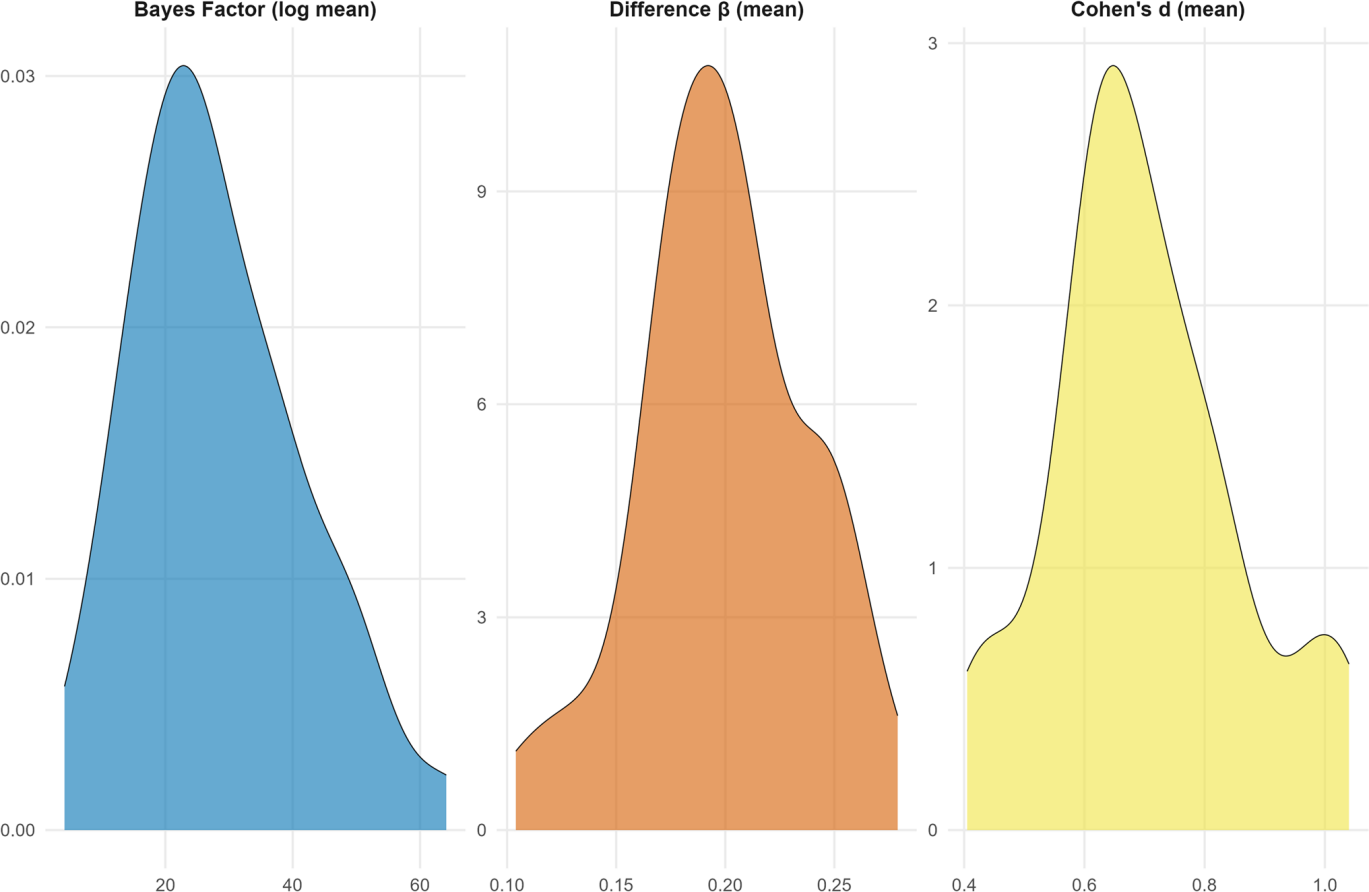
Plotted densities of three investigated parameters in a randomly sampled experiment per each simulation with 2000 iterations

The consistent positive direction of difference (beta) further signifies that the concepts have higher expressibility in gesture than in vocalizations. However, the high variance of Cohen’s *d* across all simulation types suggests that it does matter how many concepts and/or participants enter the experiment: While ‘underpowered’ simulations result in high mean Cohen’s d, simulations with more concepts and/or participants are rather conservative regarding this parameter, indicating a generally medium effect size. Figure 6 visualizes the density distribution of three main parameters: Bayes factor, difference (beta), and Cohen’s *d*. The extended descriptive summary of all descriptive parameters is provided in Table 3.

**Table 3:** Summary of the statistical parameters of the Bayesian *t* test. Note that Bayes factor values are transformed with the natural logarithm

Simulation (C, P)	BF			Difference		Cohen’s *d*	
	mean (log)	SD (log)	> 3 (in %)	mean	SD	mean	SD
Simulation 1: sampling concepts without category constraint							
Sim1 (12, 10)	11.658	7.793	95.633	0.233	0.037	0.825	0.135
Sim1 (12, 15)	18.347	11.187	98.144	0.279	0.029	1.028	0.113
Sim1 (12, 20)	25.092	14.562	99.030	0.257	0.024	1.000	0.100
Sim1 (18, 10)	17.638	9.277	99.319	0.200	0.032	0.662	0.108
Sim1 (18, 15)	27.406	13.283	99.787	0.209	0.023	0.761	0.086
Sim1 (18, 20)	37.392	17.455	99.933	0.179	0.021	0.635	0.076
Sim1 (24, 10)	23.762	10.537	99.910	0.203	0.027	0.709	0.095
Sim1 (24, 15)	36.669	15.136	99.990	0.174	0.022	0.603	0.078
Sim1 (24, 20)	49.657	19.685	99.997	0.252	0.018	0.911	0.067
Simulation 2: sampling concepts from categories actions, objects, emotions							
Sim2 (12, 10)	11.534	6.507	98.139	0.160	0.038	0.535	0.131
Sim2 (12, 15)	18.194	9.203	99.555	0.234	0.032	0.778	0.109
Sim2 (12, 20)	24.986	11.872	99.827	0.207	0.027	0.715	0.095
Sim2 (18, 10)	17.681	7.417	99.934	0.181	0.032	0.620	0.111
Sim2 (18, 15)	27.515	10.421	99.993	0.206	0.025	0.735	0.090
Sim2 (18, 20)	37.454	13.350	99.999	0.178	0.023	0.582	0.077
Sim2 (24, 10)	24.561	8.237	100	0.174	0.026	0.582	0.090
Sim2 (24, 15)	37.955	11.526	100	0.188	0.022	0.632	0.077
Sim2 (24, 20)	51.332	14.684	100	0.194	0.019	0.656	0.065
Simulation 3: sampling concepts from three randomly selected categories							
Sim3 (12, 10)	12.857	9.401	94.500	0.223	0.035	0.821	0.133
Sim3 (12, 15)	20.251	13.689	97.440	0.196	0.033	0.633	0.109
Sim3 (12, 20)	27.703	17.979	98.631	0.200	0.026	0.694	0.092
Sim3 (18, 10)	20.683	13.445	98.446	0.219	0.029	0.784	0.109
Sim3 (18, 15)	32.025	19.793	99.374	0.242	0.025	0.832	0.090
Sim3 (18, 20)	43.563	25.983	99.748	0.174	0.022	0.586	0.076
Sim3 (24, 10)	30.768	17.290	99.706	0.254	0.022	1.040	0.096
Sim3 (24, 15)	47.226	25.475	99.919	0.173	0.019	0.668	0.078
Sim3 (24, 20)	64.126	33.768	99.971	0.210	0.019	0.713	0.066
Simulation 4: sampling concepts from parts of speech nouns, adjectives, verbs							
Sim4 (12, 10)	11.447	7.832	95.125	0.205	0.037	0.735	0.138
Sim4 (12, 15)	18.111	11.306	97.917	0.255	0.028	0.964	0.113
Sim4 (12, 20)	24.740	14.743	98.810	0.123	0.025	0.429	0.089
Sim4 (18, 10)	17.353	9.308	99.135	0.197	0.032	0.666	0.110
Sim4 (18, 15)	27.117	13.454	99.804	0.176	0.024	0.619	0.086
Sim4 (18, 20)	36.848	17.595	99.905	0.245	0.021	0.846	0.078
Sim4 (24, 10)	23.338	10.538	99.919	0.178	0.026	0.619	0.092
Sim4 (24, 15)	36.046	15.113	99.985	0.155	0.023	0.500	0.075
Sim4 (24, 20)	48.794	19.753	99.995	0.136	0.020	0.437	0.065
Simulation 5: vocal-favoring category only (sanity check)							
Sim5 (10, 20)	4.166	2.958	85.879	0.104	0.303	0.405	0.119

In sum, while we observed some variation in the percentage of conclusive results – particularly for simulations with smaller numbers of observations and concepts – over 90% of simulated experiments yielded decisive evidence for gesture superiority within our 207-concept dataset. This demonstrates that the pattern of higher gestural expressibility is robust across different concept selection strategies when sampling from our concept pool. However, two things are worth noting. First, Simulation 2 (action-object-emotion selection) consistently yielded above 98% conclusive results across all conditions, including underpowered ones, suggesting that this specific category combination may favor detecting gesture superiority more readily than other selections. While this hints at potential category-driven bias in prior work using these categories, the small magnitude of differences in Bayes Factors and effect sizes across simulations prevents us from concluding that this constitutes a substantial bias. Second, Simulation 5 (emotion category only) showed the largest drop in conclusive results, falling to 85%, indicating that concept selection influences the strength of observed modality differences – particularly when sampling from domains where vocal and gestural expressibility distributions show greater overlap. Critically, our dataset shows inherent skewness toward higher gestural expressibility, with no semantic category exhibiting a median preference for vocalization over gesture. This characteristic may reflect genuine constraints on vocal modality affordances, or it may reflect limitations in our concept sampling. The validated relationship between expressibility ratings and guessability enables future research to address this question definitively: large-scale rating studies with thousands of concepts spanning more diverse semantic domains, followed by simulation analyses, would determine whether gesture superiority generalizes beyond the basic vocabulary we sampled.

## Discussion

We investigated whether self-reported expressibility ratings – participants’ intuitive judgments about their ability to communicate single concepts – can serve as validated predictors of guessability in a production experiment testing those same concepts.

Overall, Hypothesis 1 is strongly supported: expressibility boosts guessability both in binary form (perfect guess accuracy) and as a continuous measure (semantic similarity). Hypothesis 2 likewise holds: when corrections are allowed, higher expressibility reduces the number of repairs. Hypothesis 3 receives partial validation, with feedback improving correct perfect guesses but not continuous similarity. Hypothesis 4 is partially validated: vocalization alone leads to reduced guessability, whereas gesture alone appears as effective as the combined modality. Finally, we do not find evidence for Hypothesis 5 in our data, as we see no systematic bias for an inflated gesture effect caused by selective concept choice or number of concepts, and while sample size does affect statistical power, it does not systematically alter the direction of modality differences.

### Evaluating expressibility ratings against the lab experiment

Our findings provide evidence that expressibility ratings strongly predict actual communicative success across modalities. This validation has several methodological implications. Resource constraints have historically limited referential communication experiments to small concept sets and participant samples, creating significant challenges for generalizability and cross-study comparisons (Fay et al., 2014, 2022; Macuch Silva et al., 2020). Our findings suggest that expressibility ratings can serve as an efficient proxy for guessability, allowing researchers to efficiently explore much larger conceptual landscapes with greater statistical power. To boost future work, we provide a freely available comprehensive dataset on the rating of 207 concepts, 18 categories by 215 participants that can be used in investigations that try to control for expressibility ratings and more informed concept selections.

Our results show, in addition, a strong positive relationship between expressibility ratings and all three indices of communicative success – binary accuracy, cosine similarity, and the number of repetitions required. Concepts that participants rated as easy to express through gestures, vocalizations, or their combination were not only guessed more accurately, but they also required fewer communicative attempts. Thus, the ratings capture both the possibility and the efficiency of nonlinguistic communication, capturing two core dimensions of communicative effectiveness. This finding is notable given that participants’ expressibility judgments for single concepts reliably predicted actual communicative success with those concepts, demonstrating that people possess reasonably accurate intuitions about their ability to communicate basic meanings through gesture, vocalization, and their combination.

### Testing biases in experimental design

While testing the validated ratings in simulations comparing gestural and vocal expressibilities across different semantic domains did not provide evidence that the high gestural expressibility is a consequence of concept selection, we need to mention some limitations and observations. First, note that the set of concepts we use in the simulations already shows that the distribution of gestural expressibility is skewed towards higher values than that of vocal expressibility. While the 207 concepts were selected to cover basic vocabulary, they are clearly limited in capturing the diversity of meanings across a larger span of categories which would, in turn, possibly yield more equal distributions for expressibility in gesture and vocal modality. This is most evident in Simulation 5, which samples exclusively from the emotion category (i.e., a category which is generally assumed to favor the vocal modality) and in which the proportion of conclusive results (for ten concepts and 20 participants) decreased to 85%. While this does not testify for clear, existing bias in the selection of concepts, it does prove that the superiority of one modality over another depends on the overlap in the distribution. The lightweight format of ratings should encourage researchers to broaden these ratings to include a much bigger set of concepts, facilitating potential generalizability of such simulations or similar analyses.

Further, if we compare the simulation results with previous findings using a similar approach on German expressibility ratings (Kadavá et al., 2024), we can see that the strength of the evidence notably differs, with German simulations showing higher proportions of inconclusive results across all simulation types. For instance, across all simulation types with 12 concepts and 10 participants, the number of inconclusive simulations is around 10% in German, while in Dutch, this percentage decreases to 5%. This likely comes from the fact that Dutch participants seem to rate vocal expressibility slightly lower than German participants (mean 0.337 in Dutch versus 0.375 in German), making the gesture-vocal difference slightly more pronounced. One possible contributing factor could be the difference in age and background between these two groups – while German ratings were obtained from participants with a mean age of 40 years and a range between 18 and 70 years from different backgrounds, Dutch participants included mainly college students with a mean age of 20 years and a range between 18 and 68 years. This highlights how different languages or cultures (even if closely related) and other demographics can introduce bias toward one modality. Such findings demonstrate the utility of expressibility ratings that can easily accommodate very diverse samples from populations, thereby allowing researchers to study systematic patterns in expressibility caused by linguistic, cultural, generational, or other demographic factors.

### Modality differences and their theoretical implications

Our findings partially support the hypothesized hierarchy of modality effectiveness (combined > gesture > vocalization), but with an important qualification. While vocalization consistently underperformed both gesture and the combined modality across all measures, we found no reliable difference between gesture alone and the combined gesture-vocalization modality. This pattern aligns with studies highlighting the importance of gestures in novel communication systems (Fay et al., 2014, 2022); however, it must be further examined to disentangle our result from previous research showing advantages for multimodal communication over unimodal (Macuch Silva et al., 2020).

Contemporary multimodal frameworks of language (Holler, 2022; Kendon, 2017; Levinson & Holler, 2014) emphasize the inherent flexibility of human communication, with different channels offering complementary affordances depending on communicative context and content. The equivalence we observed between gesture-only and combined modalities for guessing accuracy measures demonstrates that for a single-concept referential task, gesture alone provided sufficient communicative resources. However, this does not preclude multimodal advantages in other dimensions.

Multimodal communication may offer advantages beyond accuracy that our experimental design did not capture. The combined modality potentially provides benefits in: (1) communicative efficiency and speed, as Macuch Silva et al. (2020) found shorter trial lengths for multimodal versus gesture-only communication; (2) effort distribution across channels, reducing physical demands (Kadavá et al., 2025); (3) redundancy when one channel is degraded; and (4) communicating complex meanings such as events or narratives where sign language research shows coordinated use of multiple articulators becomes essential for spatial coherence and perspective-taking (Perniss, 2007). Our task – single concrete concepts without time constraints – may not have created conditions where these multimodal advantages manifest.

Our findings demonstrate that gesture provides robust communicative effectiveness for communicating single meanings across both rating and production methodologies, while the conditions under which multimodal communication provides additional benefits beyond accuracy remain an important direction for future research.

### The role of feedback in communicative evolution

The modest improvement in binary guessability with feedback, coupled with no meaningful change in concept similarity (measured as cosine similarity), offers insights into communicative adaptation. Feedback appears to help participants converge on exact lexical matches without necessarily improving the overall semantic proximity of guesses.

This pattern suggests that feedback may primarily facilitate the alignment of specific form-meaning mappings rather than broad semantic understanding. When participants receive feedback that their guess was incorrect, they may refine their understanding of the specific target label without substantially altering their overall conceptual proximity to the target. This distinction is important for understanding the mechanisms of communicative adaptation, particularly in novel communication systems where conventions are being negotiated in real-time. This selective improvement in binary guessability but not in concept similarity despite the fixed order in feedback conditions suggests that the effect stems from convergence on exact matches, and is not a general performance gain from task familiarity.

The relatively modest effect of feedback overall may reflect the time-limited nature of our experimental interactions. Studies of longer-term communicative evolution (Kirby et al., 2008; Tamariz & Kirby, 2016) typically show stronger effects of feedback over multiple generations or extended interactions. Our single-session design likely captured only the initial stages of communicative adaptation, suggesting the need for longitudinal designs to fully understand how feedback shapes emerging communication systems over time.

### Methodological limitations and future directions

While our validation of expressibility ratings offers promising methodological advances, several limitations warrant acknowledgment. Our empirical validation was conducted within a specific experimental paradigm: whole-body movements in dyadic laboratory interactions with Dutch university students. The sensitivity of expressibility ratings to design-specific factors (seated vs. standing position, manual vs. whole-body gesture, group vs. dyadic interaction) and generalizability across populations, languages, and cultures requires systematic investigation. Our contribution is an empirical validation establishing that ratings predict guessability within this paradigm; comprehensive validation would require meta-analytic comparison across diverse experimental designs. Additionally, while rating studies standardly focus on individual lexical items (as in iconicity, age of acquisition, and sensorimotor norm databases), future research could investigate whether rating approaches extend to more complex communicative structures such as events or narratives. Communicating complex meanings involves fundamentally different processes than communicating individual lexical items (Motamedi et al., 2019; Nölle et al., 2018; Schouwstra, 2017), and sign language research demonstrates that iconicity manifests differently in lexical signs versus narrative-level depiction (Perniss, 2007; Slonimska et al., 2021, 2022). Whether expressibility ratings would predict communicative success for such complex structures remains an open empirical question.

Second, our models revealed substantial concept-level variation in the expressibility–guessability relationship, suggesting that concept characteristics (such as semantic domain, sensorimotor properties, iconicity potential) systematically influence this correspondence. While an in-depth analysis of these factors is beyond the scope of this paper, this observation aligns with research on modality-specific affordances for different types of concepts (Ortega & Özyürek, 2020; Perniss et al., 2015) and offers promising directions for future research examining when and why expressibility ratings are most predictive.

Third, our experimental design focused on one-time communicative interactions rather than extended communicative evolution. The modest effect of feedback suggests that longer-term processes involving extensive grounding and conventionalization might yield different outcomes. Longitudinal designs could help establish how expressibility ratings relate to communicative success across different timescales.

Finally, while our concept selection aimed to represent diverse semantic domains and expressibility levels, it is limited to a basic vocabulary that does not achieve equal distributions of expressibility, guessability, or more broadly, modality affordances across concepts and categories. This limitation became most evident in the simulations. While we find robust evidence for gestural superiority across different concept selection strategies, we cannot claim with certainty how much these results generalize to larger or more balanced concept sets. The expressibility distribution in our dataset shows clear skewness toward higher gestural expressibility, with no semantic category showing a median preference for vocalization over gesture. This inherent characteristic of our dataset – which may reflect genuine constraints on vocal modality or may simply reflect the particular concepts we sampled – limits our ability to test whether there exists semantic domains where vocalization systematically outperforms gesture. Future research with larger, more strategically sampled concept sets would be needed to definitively address this question. Similarly, future investigations could focus on how different concepts moderate the expressibility–guessability relationship. Certain semantic domains may show stronger or weaker correspondence between intuitive judgments and actual performance.

## Conclusion

In conclusion, our joint findings from a rating and production study validate expressibility ratings as an efficient methodological tool for studying the impact of different communicative modalities on the capacity to create and share meaning. Ratings reliably predict guessability measured both as binary accuracy and as continuous semantic similarity, predict the efficiency of communication (number of corrective attempts), and capture differences in effectiveness across modalities. Our production experiment shows that gesture and combined modalities outperform vocalization for communicating single concepts, replicating established patterns. The strong relationship between expressibility and guessability for single concepts demonstrates that people’s intuitive judgments about their ability to communicate basic meanings are accurate predictors of communicative success, even in the absence of conventional language systems. By means of a comprehensive open-access dataset including ratings of more than 200 concepts and a simulation tool taking the choice of concepts and sample size into account, we hope to contribute to future studies on language evolution.

## Data Availability

All data, scripts, and models are available in the GitHub repository: https://github.com/sarkadava/FLESH_Expressibility_vs_guessability.

## Footnotes

1 We use the term “expressibility” to refer specifically to these subjective assessments of communicative potential for individual concepts, distinct from Searle’s (1969) Principle of Expressibility in speech act theory and from “expressivity” as used in experimental semiotics to describe how signs become more distinct through interaction (Winters et al., 2015).

## Code availability

All data, scripts, and models are available in the GitHub repository: https://github.com/sarkadava/FLESH_Expressibility_vs_guessability.
